# Affordances in the home environment of children at risk of developmental delay

**DOI:** 10.1590/1984-0462/2023/41/2022104

**Published:** 2023-05-15

**Authors:** Janaína Araujo Teixeira Santos, Amanda Larissa Oliveira Lima, Letícia Dias dos Santos Silva, Fernanda da Costa Braga, Marcelo Machado Alécio, Paula Silva de Carvalho Chagas, Érica Cesário Defilipo, Aline Martins de Toledo, Paulo José Barbosa Gutierres, Kênnea Martins Almeida Ayupe

**Affiliations:** a Universidade de Brasília, Brasília, DF, Brazil.; b Bethany College, Lindsborg, KS, United State.; c Universidade Federal de Juiz de Fora, Juíz de Fora, MG, Brazil.

**Keywords:** Child development, Environment, Low income, Risk factor, Desenvolvimento infantil, Meio ambiente, Baixa renda, Fator de risco

## Abstract

**Objective:**

The aim of this study was to verify the adequacy of affordances in the home environment of children at risk of developmental delay and to identify factors associated with their frequency.

**Methods:**

The cross-sectional study included 97 families who responded to the Affordances in the Home Environment for Motor Development — Infant Scale (AHEMD-IS) for 3–18 months (n=63), or AHEMD – Self-Report (AHEMD-SR) for 18–42 months (n=34). The Mann-Whitney U test was used to identify the differences between the frequencies of affordances between the groups. Multiple linear regression was used to verify the association between the child’s sex, mother’s marital status, education, socioeconomic level, child and mother’s ages, house residents’ number, per capita income, and AHEMD scores (α=0.05).

**Results:**

The home affordances’ frequency in the AHEMD-IS ranged from less than adequate to excellent, while in the AHEMD-SR, the highest predominance was medium. The offer of stimuli in the AHEMD-IS was significantly higher. Higher socioeconomic level and house residents’ number were associated with greater affordances.

**Conclusions:**

The higher the socioeconomic level and house residents’ number, the greater the affordances in the homes of children at risk of delay. It is necessary to provide families with some alternatives to make their home environments richer in affordances that favor child development.

## RESUMO

**Objetivo:**

Verificar a adequação das oportunidades de ação no ambiente doméstico de crianças em risco de atraso no desenvolvimento e identificar fatores associados à sua frequência.

**Métodos:**

Este estudo transversal incluiu 97 famílias que responderam ao *Affordances in the Home Environment for Motor Development - Infant Scale* (AHEMD-IS) para 3–18 meses (n=63) ou o *Self Report* (AHEMD-SR) para 18–42 meses (n=34). O teste de Mann-Whitney foi utilizado para identificar as diferenças entre as frequências de oportunidades de ação entre os grupos. A regressão linear múltipla foi utilizada para verificar a associação entre o sexo da criança, o estado civil, a escolaridade e nível socioeconômico da mãe, as idades da criança e da mãe, o número de residentes da casa, a renda *per capita* e os escores do AHEMD (α=0,05).

**Resultados:**

A frequência das oportunidades de ação dos domicílios no AHEMD-IS variou de menos do que adequado a excelente; enquanto, no AHEMD-SR, a maior predominância foi de média frequência. A oferta de estímulos no AHEMD-IS foi significativamente maior. O maior nível socioeconômico e o número de moradores da casa foram associados a maiores oportunidades de ação.

**Conclusões:**

Quanto maior o nível socioeconômico e o número de moradores, maiores são as oportunidades de ação nos domicílios das crianças em risco de atraso. É necessário oferecer às famílias algumas alternativas para tornar os seus ambientes domésticos mais ricos em oportunidades de ação que favoreçam o desenvolvimento infantil.

## INTRODUCTION

Child development is an individual process that is influenced by the complex interaction between biological, personal, and environmental factors.^1,2^ The family and school are the main contexts of child development, since these are the first environments of the child’s motor, social, and mental experience.^2,3^

The capacity of a child to explore the environment is extremely important for their development and behavior.^2,4^ The first years of life are strongly influenced by the discovery of possibilities for action in different environments.^5^ Affordances are opportunities offered by the environment for individual action and, consequently, for learning and developing skills.^5^ Affordances are available in all spaces and objects; thus, as the infant is able to differentiate the environment around them, they begin to perceive the possibilities of dynamic changes within themselves.^6,7^

The home environment could have a facilitator or a barrier effect on the progression of the child’s development.^3,4,8^ The main home affordances are in the architecture of the house, physical space, and variety of toys.^4^ The literature has shown that an inadequate supply of home environmental stimuli, such as the absence of toys, is associated with delay in cognitive and social development.^9,10^ In contrast, environments that provide adequate affordances have a positive effect on neuronal development and brain connections, especially in early childhood.^8,11^

The association between child development and affordances is well established in the literature;^3,5,7,8,10–12^ however, not much is known about which factors influence the existence of affordances in the home environment of Brazilian children between 3 and 42 months. The aim of this study was to verify the adequacy of affordances in the home environment of children at risk of developmental delay and to identify factors associated with the home affordances’ frequency. We aimed to answer the following questions: What are the frequencies of affordances in Brazilian homes of infants and children at risk of development delay? Is there a difference between frequencies of affordances in the homes of infants (3–18 months) and children (18–42 months)? Which contextual factors are associated with the frequency of affordances in the homes of infants and children?

## METHOD

This is a quantitative, cross-sectional study approved by the Research Ethics Committee of the University of Brazil (CAAE: 93584218.9.0000.0030). The children were selected in public follow-up outpatient services and in the Early Education Programs of the Federal District-Brazil, between July 2019 and December 2021. The children were eligible according to the following inclusion criteria: being between 3 and 42 months of age, participating in early stimulation or follow-up programs, and presenting some risk factors, such as prematurity, for developmental delay.^8^ Children with a confirmed diagnosis of genetic or neurological diseases, such as Down Syndrome and Cerebral Palsy, were excluded. Parents who agreed to participate signed the consent form.

Information about the infants and children was collected from the medical records or a parent’s interview. The following factors were considered independent variables: child’s age and sex, maternal and paternal education, mother’s age, mother’s marital status, house residents’ number, socioeconomic level, and per capita income. The socioeconomic classification level was evaluated using the Brazilian Economic Classification Criterion (CCEB), which classifies families into levels A, B1, B2, C1, C2, and D/E.^13^ For these analysis purposes, the children were grouped into upper level (A/B1/B2), middle level (C1/C2), and lower level (D/E). For children born prematurely, the age was corrected until 24 months. The home affordances’ frequency was considered a dependent variable, classified by the Affordances in the Home Environment for Motor Development (AHEMD) questionnaire. The instrument allows a simple, fast, and effective evaluation of affordances for motor development in the family environment through a questionnaire applied to the parents. The AHEMD is subdivided into two versions, namely, the Infant Scale (AHEMD-IS) for infants aged 3–18 months, and the Self-Report (AHEMD-SR) for children aged 18–42 months. Both these questionnaires are translated into Portuguese.^14,15^ The items (questions) of the questionnaire are supplemented with illustrations for better understanding by parents and the responses are dichotomous (yes or no) or based on a Likert scale.^14,15^

The AHEMD-IS is made up of 35 questions and divided into 4 dimensions, namely, Physical Space, Variety of Stimulation, Fine-Motor Toys, and Gross-Motor Toys. The score of each dimension and the total score are calculated for the age groups of 3–11 months (26 items) and 12–18 months (35 items), allowing the classification of the environment as “less than adequate”, “moderately adequate”, “adequate”, and “excellent”. For infants aged between 3 and 11 months, the total score ranges from 0 to 49 points, while for those aged 12–18 months, the score ranges from 0 to 67 points.^14^

The AHEMD-SR is made up of 67 questions and divided into five dimensions, namely, Inside Space, Outside Space, Variety of Stimulation, Fine-Motor Toys, and Gross-Motor Toys. Three different classifications are used to evaluate the total score: “low” (up to 9 points), “average” (10–16 points), and “high” (17–20 points). To specify the five dimensions, the following classifications of the AHEMD-SR were used: “very weak”, “weak”, “good”, and “very good”. To generate the results, the answers to all items are entered into an online calculator, which automatically provides the final score, according to the child’s age.^15,16^

For this study, the participants were classified into two groups according to the age and questionnaire applied; the AHEMD-IS group was composed of infants aged 3–18 months and the AHEMD-SR group was composed of children aged 18–42 months.

Mean, standard deviation, and/or frequency values were calculated for all variables, and the qualitative variables were transformed into ordinals. The Mann-Whitney U test was used to verify the differences in the independent variables between the two groups. The chi-square test was used to verify the home affordances’ frequency classification differences in each dimension and between the total score of each group.

In this study, the four AHEMD-IS final home affordances’ frequency classifications were reorganized into three classifications in order to correspond to the AHEMD-SR, as follows: “adequate” and “excellent” AHEMD-IS classifications corresponding to the “high” AHEMD-SR classification; “moderately adequate” AHEMD-IS classification corresponding to the “average” AHEMD-SR classification; and “less than adequate” AHEMD-IS classification corresponding to the “low” AHEMD-SR classification. The Mann-Whitney U test was used to identify the differences between the final home affordances’ total score between the two groups (α=0.05). The standardized effect size was calculated by the equation (Z/√N), and the results were interpreted as small (0.10–0.20), medium (0.30–0.40), and large (≥0.50).^17^

To verify the association between the home affordances’ frequency and the independent variables, the total score of each instrument was used. Initially, the association with the categorical independent variables (child’s sex, mother’s marital status, maternal and paternal education, and socioeconomic level) was verified by Kendall’s Tau-b test. The correlation with the quantitative independent variables (child’s age and mother’s age, house residents’ number, and per capita income) was shown by the Spearman’s correlation test, classified as weak correlation (0.10>r>0.30), moderate correlation (0.30>r>0.50), and strong correlation (r>0.50).^17^ Later, confirmatory analyses were performed using a multiple linear regression model (stepwise) to verify the association between the scores of each instrument with the variables that showed a significant relation in the previous correlation analysis (α=0.05). All data were analyzed using the Statistical Package for Social Sciences (SPSS)^®^ version 23.0.

## RESULTS

One hundred families were eligible to participate during the study period, of which 97 answered the questionnaire, 63 parents of infants aged 3–18 months (AHEMD-IS group), and 34 parents of children aged 18–42 months (AHEMD-SR group). The characteristics of the infants, children, and their families are shown in Table 1. There was no difference between these two groups when considering the independent variables of the study, except for maternal education, which was significantly higher in the AHEMD-SR group.

**Table 1.** Characteristics of the study participants and their families.

Study variables	AHEMD-IS n=63	AHEMD-SR n=34	Total sample n=97
Child’s age (months) (SD)	8.6 (±3.9)	31.4 (±8.1)	29.8 (±7.1)*
Sex n (%)			
Male	37 (58.7%)	19 (55.9%)	56 (57.7%)
Socioeconomic level n (%)			
Lower	31 (49.2%)	16 (47.1%)	47 (48.5%)
Middle	29 (46.0%)	15 (44.1%)	44 (45.4%)
High	3 (5.0%)	3 (8.8%)	6 (6.2%)
Per capita income (SD)	651.12 (±569.17)	671.68 (±474.91)	658.00 (±535.58)
Mother’s age (years) (SD)	31.2 (±7.3)	27.2 (±5.8)	29.8 (±7.1)
Mother’s marital status n (%)			
Married/stable marriage	50 (79.0%)	28 (82.4%)	78 (80.4%)
Single/divorced	13 (21.0%)	6 (17.6%)	19 (19.6%)
Maternal education (%)			
Up to 5 years	10 (16.0%)	2 (5.9%)*	12(12.6%)
6 to 9 years	13 (21.0%)	5 (14.7%)	18 (18.5%)
10 to 12 years	30 (48.0%)	17 (50.0%)	47 (48.4%)
>12 years	10 (16.0%)	10 (29.4%)*	20 (20.6%)
Paternal education (%)			
Not literate	3 (5.0%)	1 (2.9%)	4 (4.1%)
Up to 5 years	7 (11.0%)	5 (14.7%)	12 (12.3%)
6-9 years	12 (19.0%)	2 (5.9%)	14 (36.1%)
10-12 years	28 (44.0%)	16 (47.1%)	44 (45.5%)
>12 years	10 (16.0%)	8 (23.5%)	18 (18.6%)
House residents’ number (SD)	4 (±0.86)	4 (±0.98)	3.9 (±0.90)

AHEMD: affordances in the home environment for motor development; IS: infant scale; SR: self-report; n: number of subjects; SD: standard deviation; %: frequency. *p<0.05.

Table 2 shows the results of home affordances’ frequency classifications of the AHEMD-IS group. In the Physical Space dimension, the most frequent classification was “moderately adequate”; and in the Variety of Stimulation dimension, the most frequent classification was “excellent”; in the Fine-Motor Toys dimension, the most frequent classification was “adequate” (p<0.05). In the Gross-Motor Toys dimension and in the total score, there was not a significant most frequent home affordances’ frequency classification (p>0.05).

**Table 2.** Home affordances’ frequency classification of the Affordances in the Home Environment for Motor Development for Infant Scale group dimensions and total score.

Dimensions	Less than adequate (%)	Moderately adequate (%)	Adequate (%)	Excellent (%)
Physical space*	28.6	46.0	20.6	4.8
Variety of stimulation*	9.5	20.6	31.7	38.1
Gross-motor toys	28.6	31.7	23.8	15.9
Fine-motor toys*	31.7	11.1	33.3	23.8
Total score	22.0	25.4	25.4	27.0

%: frequency. *p<0.05.

Table 3 shows the results of home affordances’ frequency classifications of the AHEMD-SR group. In the Variety of Stimulation and Inside Space dimensions, the most frequent classification was “very good”; in the Outside Space dimension, the most frequent classification was “weak”; and in the Fine-Motor and Gross-Motor Toys dimensions, the most frequent classification was “very weak” (p<0.05). In the total score, the majority (79.4%) of home affordances’ frequency was classified as “average” and none was classified as “high” (p<0.05). In the between-groups comparison between AHEMD-IS and AHEMD-SR, a significant difference was identified between the final home affordances’ frequency classification (p≤0.001), demonstrating a positive result for the AHEMD-IS group, with a moderate effect size (d=0.35).

**Table 3.** Home affordances’ frequency classification of the Affordances in the Home Environment for Self-Motor Development Report group dimensions and total score.

Dimensions	Very weak (%)	Weak (%)	Good (%)	Very good (%)
Variety of stimulation*	2.9	5.9	14.7	76.5
Outside space*	20.6	44.1	26.5	8.8
Inside space*	8.8	11.8	14.7	64.7
Gross-motor toys*	70.6	26.5	2.9	0
Fine-motor toys*	85.3	8.8	0	5.9

%: frequency. *p<0.05.

The association analysis shows a significant moderate positive correlation between the total AHEMD-IS score (home affordances’ frequency) and the child’s age (r=0.407; p≤0.05), socioeconomic level (r=0.441; p≤0.05), and per capita income (r=0.342; p≤0.05), and a significant moderate negative correlation with maternal education (r=−0.306; p≤0.05). In the AHEMD-SR group, it was found a significant strong positive correlation between the total score and socioeconomic level (r=0.609; p≤0.05), a significant moderate positive correlation with the house residents’ number (r=0.300; p≤0.05), and a significant weak negative correlation with child’s age (r=−0.286; p≤0.05). The other variables presented very weak correlations with the dependent variables.

Table 4 shows the results of linear regression analyses for the two groups separately. The results for AHEMD-IS demonstrated a composite predictor model with two variables (socioeconomic level and child’s age) that, together, explained 31.2% of the score variance. It is noteworthy that the socioeconomic level showed the highest explanatory power (19.4%). The set of variables demonstrated that the higher the socioeconomic level (β=0.387; p≤0.001) and child’s age (β=0.346; p≤0.002), the higher the AHEMD-IS final score. Regarding the AHEMD-SR, the regression model explained 49.7% with two variables (socioeconomic level and house residents’ number), with the socioeconomic level demonstrating the highest explanatory power (41.92%). Therefore, the higher the economic level of the family (β=0.659; p≤0.001) and house residents’ number (β=0.261; p≤0.003), the higher the AHEMD-SR final score.

**Table 4.** Multiple linear regression analysis (stepwise) between the independent variables and the Affordances in the Home Environment for Motor Development for Infant Scale and Affordances in the Home Environment for Motor Development for Self-Report groups total score.

Variables	R2	B	SE	β	t	p-value
AHEMD-IS						
Socioeconomic level	0.194	6.050	1.696	0.387	3.567	0.001
Child’s age	0.312	0.805	0.252	0.346	3.195	0.002
AHEMD-SR						
Socioeconomic level	0.419	2.045	0.396	0.659	5.168	<0.001
House resident number	0.497	0.570	0.261	0.279	2.184	0.003

AHEMD: Affordances in the Home Environment for Motor Development; IS: Infant Scale; SR: Self-Report; SE: standard error; β: standardized beta coefficient; t-distribution: Student’s t

## DISCUSSION

This study identified that the home affordances’ frequency for infants aged 3–18 months ranged from “less than adequate” to “excellent”, and for children aged 18–42 months, the highest frequency of affordances was “medium”. The affordances’ frequency in the younger infant’s homes was significantly higher than in the children’s homes. This study also identified that the higher the socioeconomic level and house residents’ number, the greater the affordances in the homes of Brazilian infants and children at risk of developmental delay.

In this study, the home affordances’ frequencies of the total score of the AHEMD-IS group were similarly classified between “less than adequate” and “excellent,” while previous studies had shown frequencies between “less than adequate” and “moderate”.^3,4,8,11,18,19^ Another Brazilian study, also including a population of families of children with psychosocial risk indicators, identified a prevalence of the classification “less than adequate” for the home affordances’ frequency on AHEMD-IS.^19^ The families participating in the present study, although predominantly of low economic status, participate in developmental follow-up programs where they are informed about early stimulation, which may justify the better result of the present study in relation to the previous.

In the AHEMD-IS group, the dimension Physical Space presented a higher prevalence of worst classifications, showing that the physical and architectural structure of the homes is not adequate for the motor development of the infants studied. This result is similar to another study carried out in southeastern Brazil, which evaluated affordances in 77 homes of infants with hearing impairment and found that the opportunities related to Physical Space in the households were inadequate.^18^ An environment with low-quality stimuli and inadequate architectural structure may be related to several factors, especially the lack of financial resources needed for better housing conditions.^3,4^

The home affordances’ frequencies of the AHEMD-SR group were mostly inadequate, highlighting that the Fine-Motor and Gross-Motor Toys dimensions showed a higher predominance of the “very weak” classification. These results corroborate with other studies that also identified a low supply of toys in the homes of children at the same age.^10,20,21,22^ In contrast, a study conducted in the southern region of Brazil identified good availability of Gross-Motor Toys, demonstrating that better financial resources facilitate the availability of toys at home.^6^

We also found, in the AHEMD-SR group, a predominance of the “weak” classification in the External Spaces dimension, while the Internal Spaces dimension presented a higher prevalence of the “very good” classification. This divergence is in line with other studies, which indicate a “weak” classification of the Outside Spaces and better adequacy of the Inside Spaces, in addition to a “very good” classification of the Variety of Stimulation dimension.^6,20,23,24,25^ In Brazil, low-income families tend to live in small houses or apartments without outdoor areas, which justifies these results. In this sense, it is important that low-income countries offer public recreational spaces, such as squares and parks, to contribute to the adequate development of children living in the community.^26^

In between-groups comparison, this study identified a significantly higher home affordances’ frequency in the AHEMD-IS group. We did not identify other studies in the literature that compared the affordances’ frequency between the two instruments (AHEMD-IS and AHEMD-SR). It is possible that this difference in affordances between the groups is related to the characteristic of the instrument, since the AHEMD-IS items are less complex, due to the age group. This result may also be associated with the period of maternity leave, in which mothers are involved in baby care and spend more time with them, raising the AHEMD-IS score compared to the AHEMD-SR score.^4,19,27^

Regarding the factors associated with the home affordances’ frequency, it was identified that the lower the socioeconomic level, the lower the affordances’ frequency in both groups. Family income is recognized as a determining factor for greater adequacy of the home environment and for child development.^4,7,8,20,23,26,28^ The socioeconomic level is related to both the quality of the indoor and outdoor environment, as well as the offer of materials and toys.^9^ In addition to socioeconomic status, in the AHEMD-IS group, older infant ages were also associated with a higher home affordances’ frequency. A longitudinal study performed in Brazil evaluated the affordances’ frequency in the homes of infants at 3, 6, 9, and 12 months of age and found that the affordances in the Variety of Stimulation domain were better in the older ages.^3^ However, the authors believe that in the reassessment by the AHEMD-IS, the parents identified the need for the acquisition of toys corresponding to each age group.^3^

In the AHEMD-SR group, a greater number of family members living in the same home were also associated with a greater home affordances’ frequency. Similar results were found in a study conducted in the southern region of Brazil that identified an association between a greater number of adults and a higher Variety of Stimulation.^23^ In addition, a study conducted in Portugal identified that the Fine-Motor Toys dimension is the most sensitive to the number of children in the house.^11^ Residences with only one child tend to offer less stimuli for the child development, since during their development, the child tries to reproduce the movements of other nearby children, such as brothers.^23^

Interventions focused on the environment with family participation have shown evidence of a positive effect on child development, so they should be implemented early in the care of children at risk of motor delay.^29,30^ In this study, the variables that were more related to the low home affordances’ frequency (low socioeconomic level and house residents’ number) are not manageable by health professionals. However, many other characteristics of the home environment can be modified so that it becomes rich in affordances.^29,30^ Therapists can guide families on how to organize the available space in the home so that the child can better explore the environment.^29,30^ Families should be oriented to develop, together with the child, toys made of low-cost materials and, thus, spend more time with the child.^29^

Considering the limitations of the study, we highlight the lack of homogeneity between the quantity of children between the two age groups studied, as the number of participants in the AHEMD-IS group was higher than in the AHEMD-SR group. In addition, as we used a convenience sample, without calculating the sample size, it is possible that the number of participants was not enough to demonstrate all existing correlations. We point out the difference between the two instruments in relation to scores and classifications, which hinders the comparisons between the two groups, making adaptations necessary.

In conclusion, the homes of Brazilian children at risk of developmental delay have an unsatisfactory frequency of affordances, especially in physical space and availability of toys. The frequency of affordances in the house is related to the child’s age, the house residents’ number, and the socioeconomic level. It is necessary for therapists to help families to make the home environment richer in affordances in order to favor child development.

## Data Availability

The database that originated the article is available with the corresponding author.
